# Stage-Rocked Electron Channeling for Crystal Orientation Mapping

**DOI:** 10.1038/s41598-018-23413-3

**Published:** 2018-03-26

**Authors:** Karl A. Hujsak, Benjamin D. Myers, Jann Grovogui, Vinayak P. Dravid

**Affiliations:** 1 Department of Materials Science and Engineering, Northwestern University, Evanston, IL 60208 United States; 2 NUANCE Center, Northwestern University, Evanston, IL 60208 United States

## Abstract

Microstructural analysis by crystal orientation mapping of bulk functional materials is an essential and routine operation in the engineering of material properties. Far and away the most successfully employed technique, Electron Backscattered Diffraction (EBSD), provides high spatial resolution information at the cost of limited angular resolution and a distorted imaging condition. In this work, we demonstrate a stage-rocked electron channeling approach as a low-cost orientation mapping alternative to EBSD. This is accomplished by automated electron channeling contrast imaging (ECCI) as the microscope stage physically tilts/rotates a sample through a reduced hemisphere of orientations followed by computational reconstruction of electron channeling patterns (ECP). Referred to as Orientation Mapping by Electron Channeling (OMEC), our method offers advantages in terms of local defect analysis, as it combines the advantages of selected area ECP (SACP) and ECCI. We also illustrate dynamic or “adaptive” sampling schemes to increase the throughput of the technique. Finally, we discuss the implications for sample analysis in which large 3D maps of ECCI images can be routinely constructed of challenging crystalline samples. As an electron channeling-based approach to orientation mapping, OMEC may open new routes to characterize crystalline materials with high angular and spatial resolution.

## Introduction

A plethora of materials properties and phenomena revolve around the Neumann principle, which relates the innate crystal symmetry to the physical properties. In this context, crystal and phase orientation are crucial microstructural parameters that influence the mechanical, electrical, and optical properties of structures and devices. Mapping orientation across large areas (up to mm^2^) of bulk samples with high spatial resolution (10’s of nm) allows fundamental exploration of the relationship between material structure, properties, and performance. Electron backscatter diffraction (EBSD) has become the preeminent technique for performing routine orientation mapping in conventional scanning electron microscopes (SEMs)^1^. In EBSD, an electron probe scatters incoherently within a sample volume, leading to backscattered electrons (BSE) which undergo Bragg diffraction from the crystalline planes in the sample. The beam is scanned across the sample surface and at each grid point over a region of interest, the ‘cones’ of diffracted electrons are intercepted by a 2D electron detector to form an electron backscatter pattern (EBSP). Leveraging advanced detectors and computation, modern EBSD systems allow the automated acquisition and indexing of EBSP’s at speeds of more than 1500 patterns per second^2^. Nevertheless, EBSD requires a significant investment in additional hardware and suffers from limitations including image/scan distortion^3^ due to the highly tilted (typically 70 degrees) sample. Spatial resolution in standard EBSD is non-uniform (i.e different in lateral and longitudinal directions)^4^ and on the order of 20 nm while the precision of angular measurements is on the order of 1 degree^5–7^.

Diffraction contrast is also present in secondary electron (SE II & III) and BSE images and depends on the orientation of crystal planes in the sample relative to the incident electron probe. This is called electron channeling contrast and can be described by the superposition of Bloch waves that gives rise to a BSE intensity that is highly dependent on the crystal lattice orientation^8^. Recording the BSE intensity as a function of beam-specimen angle results in the formation of Electron Channeling Patterns (ECP). In practice, ECPs have been acquired by low magnification imaging of large single-crystal regions (beam rocking due to divergence in raster scan)^9^, by stage rocking with a fixed beam^8^, or by rocking the beam about a fixed point to generate a selected area electron channeling pattern (SACP)^10^.

Compared to EBSD, ECPs provide the same orientation information without the geometric distortion that comes from intercepting a three-dimensional signal from a tilted specimen onto a non-perpendicular plane^3^. Since the angles between bands are used to precisely calculate orientations, indexing can be limited by the accuracy of the forward geometric model used to account for this complex distortion. ECPs also provide high angular resolution information, provided a small beam convergence angle (<3 mrad)^8^. They have also been shown to provide strain information *via* higher-order Laue zone (HOLZ) lines^11,12^. Strain analysis by EBSD is ultimately limited by the pixel resolution of the detector, although high-resolution EBSD can provide improved angular resolution by including additional image post-processing steps^5,13^.

SACP is the most common method for ECP collection, but it requires specialized electron optics for beam rocking, which limits broad adoption of this technique. In addition, it suffers from poor spatial resolution (~500 nm) and limited angular field of view (FOV) (typically 10 degrees)^14^ due to spherical aberration at large beam tilts^15^. Historically, individual ECPs were indexed by comparing them to physical ECP-maps. These maps were laboriously constructed by synthesizing hemi-spherical single-crystal samples^8^, or fabricating dozens of uniquely oriented single crystal specimens and collecting many SACPs^16^. While useful for a limited number of simple crystal systems, these tasks were not practical for many material systems with lower symmetry or multiple phases. Methods to overcome the limited FOV and spatial resolution of SACPs, such as dictionary based indexing^17^ and the use of spherical aberration correction^18^, result in long computation times or large cost.

The related technique of electron channeling contrast imaging (ECCI) has been intensely explored for the imaging of local defects in the SEM. By mapping the local orientation and texture using EBSD (or less commonly SACP) the specimen can be tilted into a “two-beam” condition^19^. By capturing BSE images at several conditions, the Burger’s vectors and other defect parameters can be calculated without the destructive sample preparation that would be required to generate equivalent information in Transmission Electron Microscopy^20^. Several reports have analyzed dislocation and twin structures in metallic films and surfaces using this method^21–23^.

In early reports of electron channeling patterns, it was noted that a stage-rocked system with a fixed beam orientation could produce identical information to a beam-rocked ECP^24^. Early attempts at producing an accurate tilt/rotation stage were limited by the lack of computational control and could only maintain a consistent beam position within a micrometer at best^25^. More recently, techniques have been developed using ion channeling contrast in images taken at different beam-sample angles for orientation mapping by matching changes in contrast to theory/simulation^26–29^. While ion channeling contrast is extremely useful, the critical angles for ion channeling are quite large compared to electron channeling, potentially producing less accurate orientation indexing^30^. The recent electron channeling approach describe by Lafond, *et al*. generates orientation maps based on ECCIs from multiple sample orientations^31^. However, their approach does not generate full ECPs and is likely limited to orientation mapping of high symmetry systems.

**Figure 1:**
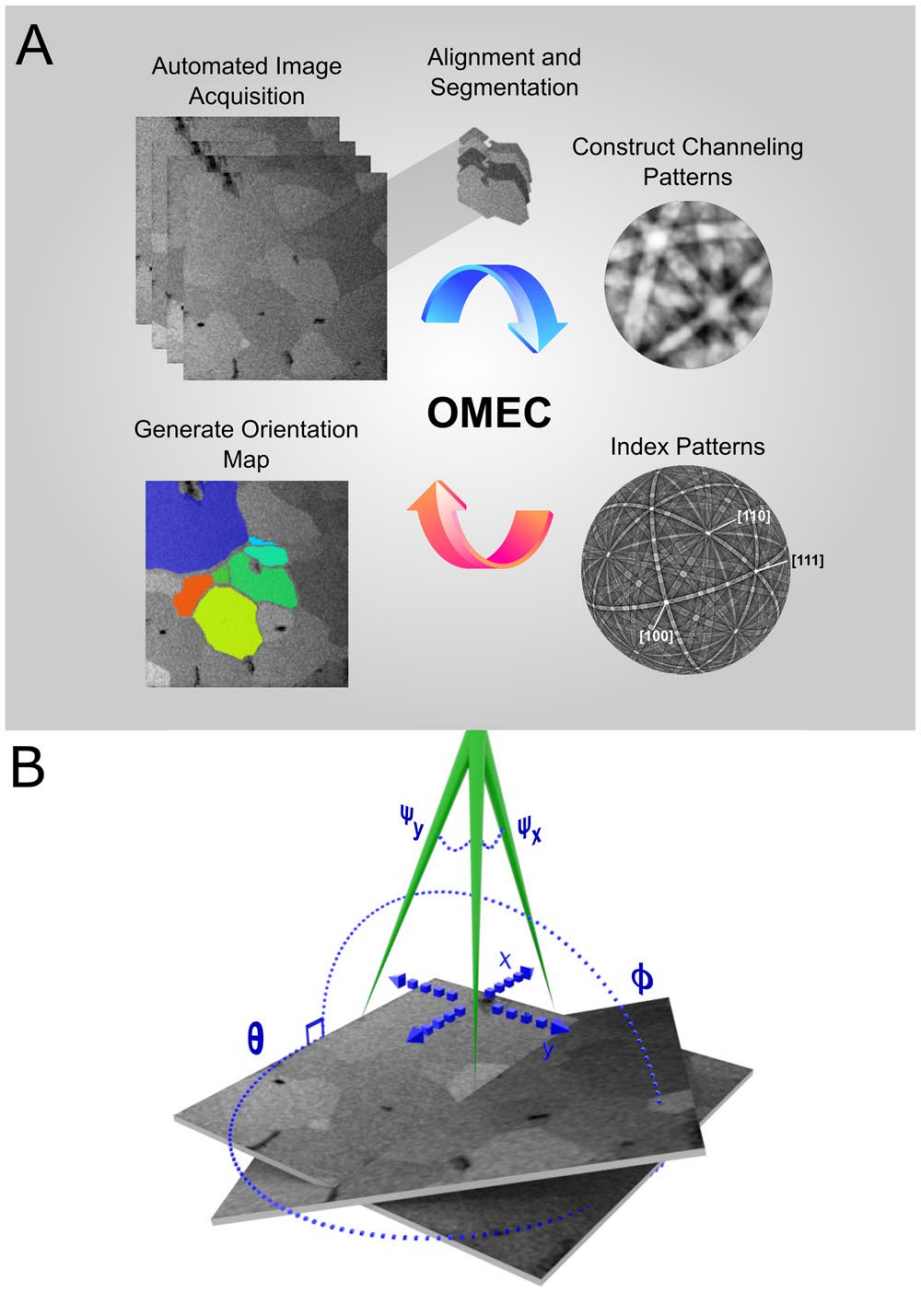
(**A**) Workflow of the proposed Orientation Mapping by Electron Channeling (OMEC) method. First, an automated image acquisition scheme acquires a tilt/rotation series of BSE images from the same relative sample area. This entire image stack is then aligned relative to each other, allowing a complete ECP to be extracted at each pixel. These channeling patterns can then be segmented or clustered (supervised vs unsupervised) into common orientations and then indexed through conventional methods. Finally, a full orientation map can be generated for the region of interest. (**B**) The variables describing various operations in the OMEC method are established, including the fixed tilt angle (φ) about a fixed axis and the in-plane rotation angle (θ). As the electron beam rasters across a fixed grid of pixels on a fixed plane defined by x,y it experiences a tilt along these axes parameterized by ψ_x_ and ψ_y_.

In the following, we demonstrate a system combining the advantages of ECCI and SACP, which enables large area mapping of crystal orientation by recording full BSE ECCIs at many different stage orientations. This is accomplished by automated stage control and image acquisition followed by computational image alignment as seen in Figure 1. This alignment corrects for any image shift, rotation or distortion and generates an image stack from which spatially resolved channeling patterns are produced. The resulting ECPs have a high angular FOV, rendering them suitable for indexing by standard routines as conventionally applied in EBSD. The resulting technique, orientation mapping by electron channeling (OMEC), results in a very cost-effective orientation mapping solution with the high angular resolution and contrast of traditional ECP without any beam-tilt induced spherical aberration.

In the first section of this paper, we examine a single-crystal silicon (100) sample and describe the hybrid stage/beam-rocking ECP that results due to the combination of stage rocking and beam rocking (due to raster scanning). As no image alignment is necessary for a single-crystal sample, the angular range and accuracy of the stage-rocked channeling system is demonstrated. By taking advantage of the spatial information in each ECCI for a polycrystalline sample, we then show how precise image alignment can be used to correct spatial distortions that occur during stage tilt/rotation to produce a high quality ECP for each grain of a PbSe/GeSe thermoelectric material. Finally, we show how the acquisition speed of the technique can be improved by sampling a reduced set of critical tilts/rotations on-the-fly with dynamic sampling, using redundant information to “synthesize” less critical ECPs.

## Results and Discussion

### Single-Crystal Channeling Patterns

**Figure 2:**
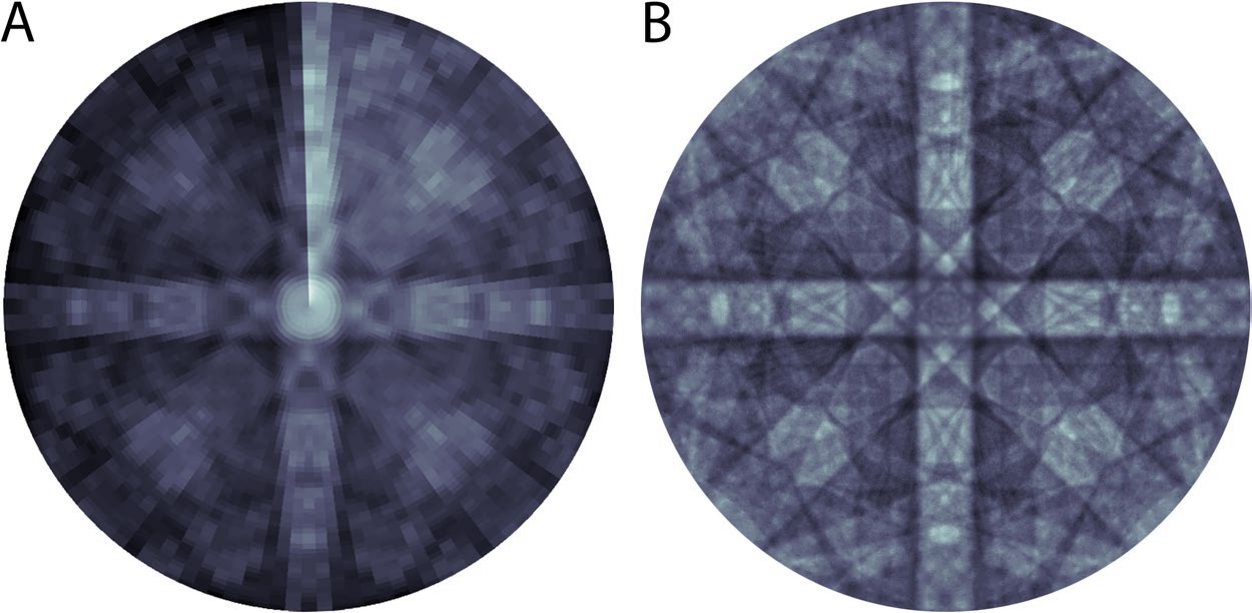
(**A**) Orthographic projection of a stage-rocked ECP reconstructed from the average contrast values in 7200 ECCIs at different orientations from a single-crystal silicon (100) sample. (**B**) Orthographic projection of a hybrid stage- and beam-rocked ECP reconstructed by binning each ECCI and correcting for beam divergence due to scanning. Corrections are also applied to adjust for systematic contrast variations (background subtraction and beam contamination correction).

To construct an ECP by stage rocking, backscattered electron (BSE) images are acquired at a series of specified tilt (φ) and rotation (θ) positions as described in Fig. 1B. These sample positions are relative to the sample surface normal and an arbitrary starting rotation position (typically registered to a relevant sample feature – in this case, the cleaved edge of the silicon wafer). A stage-rocking ECP can then be reconstructed by projecting the average contrast from each image onto a spherical surface as shown in Fig. 2A for a single-crystal silicon (100) sample. This raw ECP clearly shows the Kikuchi-like bands indicating the nominal (100) sample orientation. These data are collected on a standard commercial instrument with the capability for both eucentric tilt and rotation. However, several compromises are made in order to produce these data on such an instrument. First, the through-the-lens detector (TLD) is operated in BSE mode without the immersion field due to the high tilt range required for data collection. The TLD operated in this condition has very low sensitivity, thus an electron beam current of 1.4 nA is required to achieve sufficient signal intensity. This high beam current and the short working distance for eucentric tilting result in a relatively large beam convergence angle, which likely limits the ultimate angular resolution for electron channeling in these experiments. Further, the high beam current and long data collection time result in surface contamination that produce the discontinuity in the ECP contrast in Fig. 2A.

Despite this, we can ameliorate these issues by a few key post-processing steps. First, we remove a systematic background intensity distribution in each image, which is presumably due to geometric factors influencing detection efficiency. Second, we correct for beam divergence due to raster scanning, which lowers the angular resolution in the raw data. As the electron beam scans over the sample surface, its angle with respect to the initial sample normal is modified by a divergence angle from the scan coils, parameterized by ψ_x_ and ψ_y_. This divergence angle, and consequently the true sample-beam angle, depends on the beam position, working distance, and details of the specific electron-optical scanning system on the SEM. Thus, every pixel experiences a slightly modified angle as the scan coils tilt the beam to record a full rastered image. We can take advantage of this extra information to improve the overall angular resolution of the ECP. To accomplish this, each raw image is binned to 16 × 16 pixels, which are mapped to slightly shifted locations on the 3D orientation sphere according to the mean divergence angle for each region. This results in a 256-fold increase in the number of orientation values in the final data set – the optimal degree of binning is somewhat arbitrary and depends on collection conditions. Finally, we correct for sample contamination due to the high beam current by fitting a 3^rd^ order polynomial according to the acquisition order. As the data after divergence correction overlap in some regions of the ECP, the final ECP is plotted using grid-cell averaging. By this method, the raw stage-rocked ECP (with 7200 orientation values) is expanded to a hybrid beam/stage-rocking ECP with more than 1.8 million orientation values as shown in Fig. 2B. This results in a significant improvement in the ECP angular resolution compared to the raw data, particularly visible at the higher tilt range where data are more sparsely acquired.

### Polycrystalline Orientation Mapping

Next, we consider the task of reconstructing ECPs and generating orientation maps for a polycrystalline sample. In such a sample, the crystal orientation can change abruptly at grain boundaries, leading to very different BSE yield between two potentially adjacent pixels. Therefore, it’s crucial to ensure that pixels on either side of the boundary are correctly aligned over the course of thousands of images. For systems with many dozens of grains or where one wishes to study the change in orientation near an interface, this spatial correspondence between images becomes even more important. Briefly, contrast invariant points of correspondence are found using feature transforms. These points are then used to define a homography matrix, which accounts for the three-dimensional scaling/distortion that can occur as the object tilts around a eucentric point from a fixed perspective. This homography matrix is calculated and applied for each image in sequence, resulting in spatial correspondence through the ECCI BSE stack (see Materials & Methods for more detail).

To test our method, we employed a polycrystalline PbSe-12%GeSe crystal, a material family which has engendered much interest in the thermoelectric community^32^. Controlling microstructure and crystalline defects in these materials to reduce their thermal conductivity has received much attention, as the thermoelectric figure of merit is inversely proportional to the thermal conductivity. A set of tilted and rotated BSE images was collected in the same fashion as the silicon sample, with a maximum tilt angle of 35 degrees, a step size of 0.5 degrees, and a rotation step of 2 degrees. This resulted in 12,600 distinct BSE ECCI images, which were aligned and background corrected onto a common coordinate frame. ECPs were generated for each pixel in the aligned images as is standard practice in ECP or EBSD mapping. The patterns of each pixel contained in a grain were then integrated to form a very high contrast channeling pattern.

**Figure 3:**
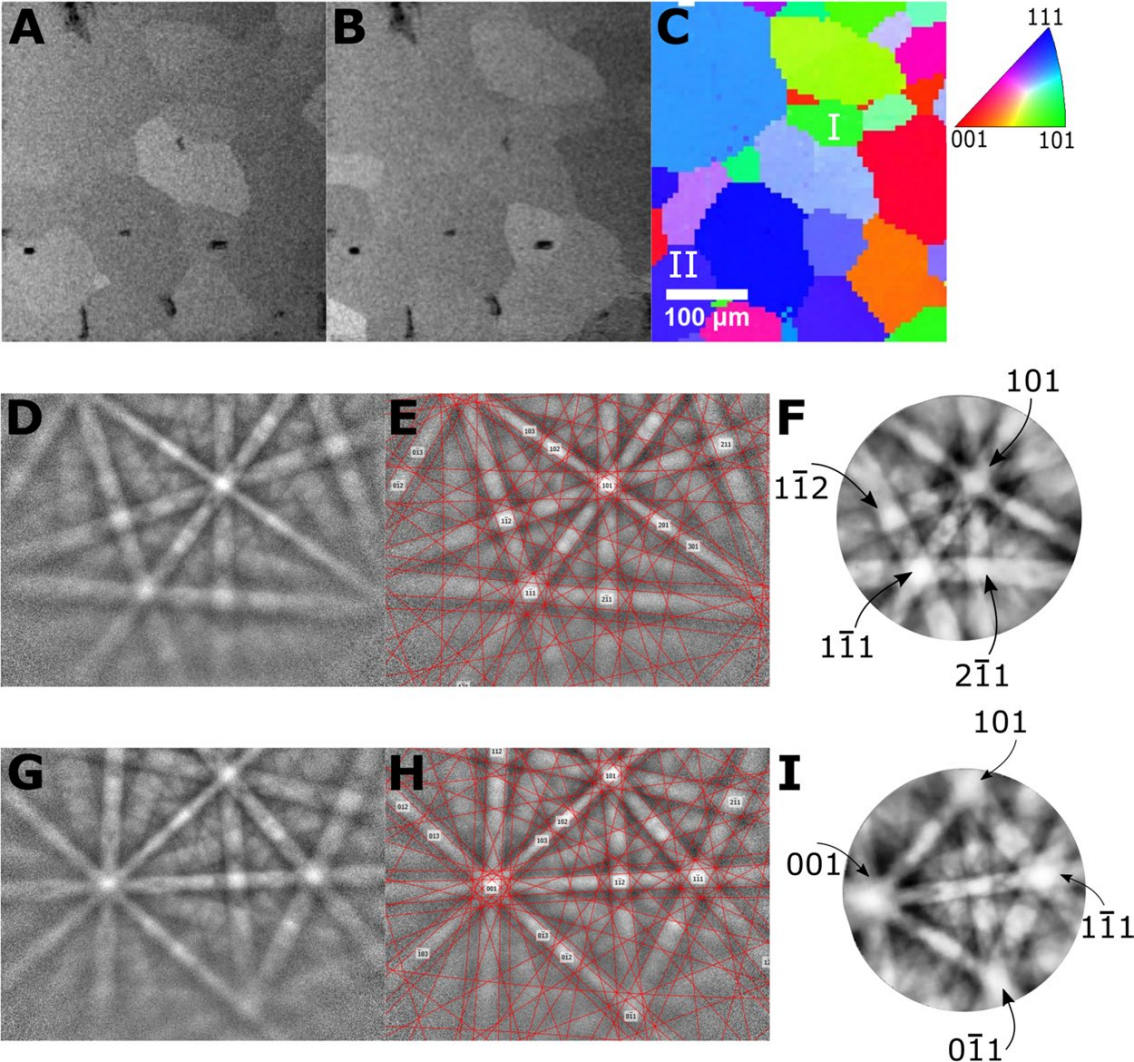
Comparison between conventional EBSD and OMEC ECPs on the same sample: (**A**,**B**) Aligned and perspective corrected BSE images of the same PbSe-GeSe crystal collected at two different orientations (A: 0 degree tilt, 0 degree rotation, B: 6 degree tilt and 0 degree rotation). (**C**) Z-axis-referenced inverse pole figure (IPFZ) map of the same area displaying the relative orientations of the member grains as indexed by EBSD. Grains I and II are labeled for the following two rows of the figure. (**D**–**F**) Representative EBSP, indexed pattern, and OMEC ECP from the grain labeled I in the IPFZ map. **(G**–**I**) A second set of EBSP, indexed pattern, and OMEC ECP for the grain labeled II in the above IPFZ map. Strong agreement between the ECP and EBSP is seen. Note that in order to assist in identifying correlation between the EBSPs and the OMEC ECPs we have chosen to project the ECPs stereographically (equiangular constructions).

Representative aligned BSE images from the total tilt series are displayed in Fig. 3, along with an EBSD map collected from the same area. As can be seen in the two BSE images, different grains light up or fade according to the diffraction condition and the angle between the beam and crystal lattice. Orientation agreement between the electron backscatter patterns (EBSPs) and the OMEC ECPs can be seen in Fig. 3, also showing the higher angular resolution and contrast inherent to channeling pattern imaging due to user control over the angular sampling with the tilt/rotation step size. The agreement between bands and their angles between the EBSPs and the ECPs is not surprising considering an *approximately* reciprocal mechanism^33^ is responsible for generation of the signal in both imaging modalities. When indexing patterns, we are interested in the relative angles between bands, and the ability to project a true equiangular construction for the ECP enables simpler indexing as compared to indexing methods for EBSD, which must account for geometric distortion. Since the ECPs are intrinsically three-dimensional data, they can be projected in many different fashions to assist interpretation.

**Figure 4:**
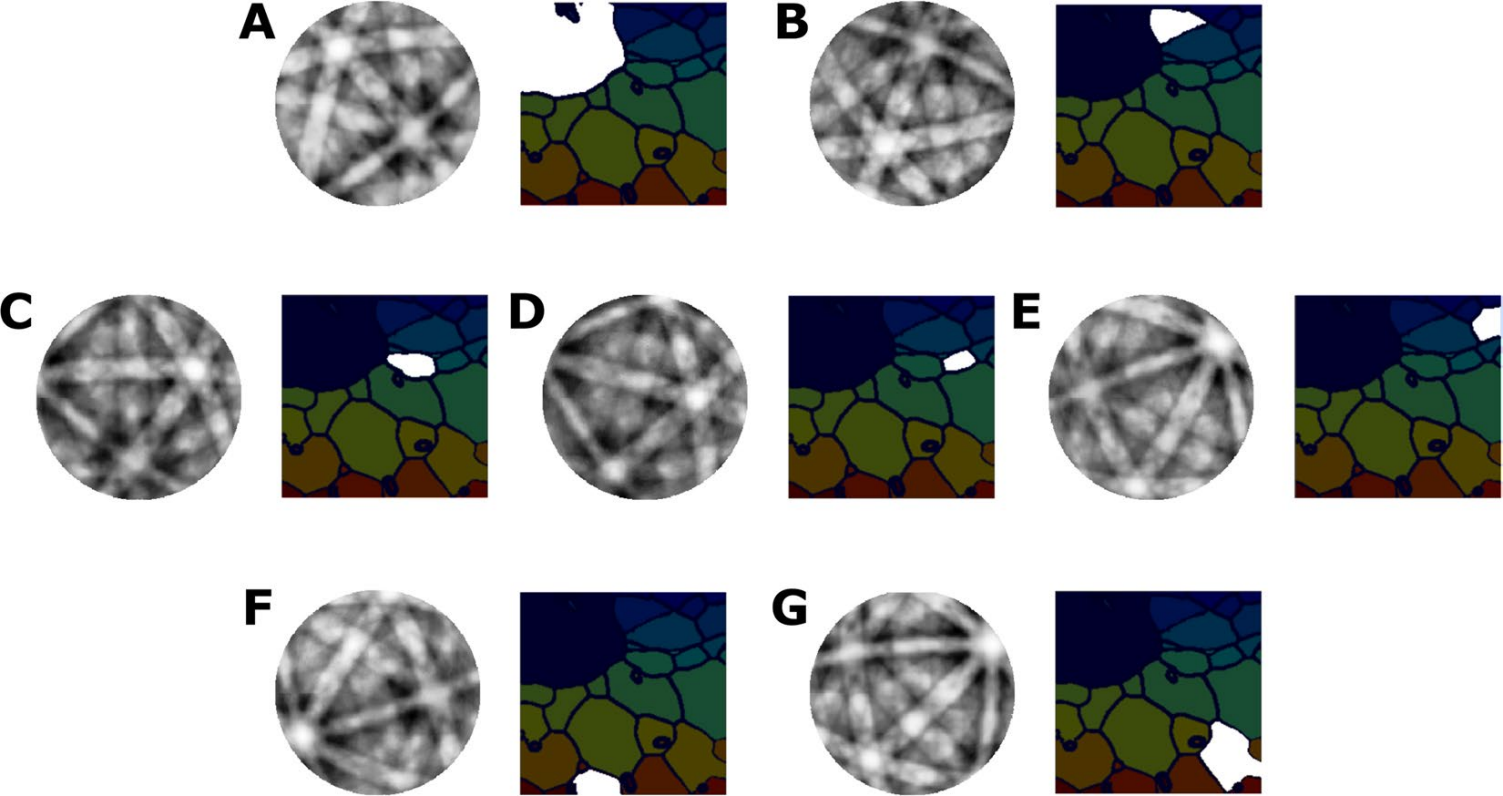
A set of orthographically projected channeling patterns for the grains displayed is shown. The true data for each grain is a real 3D dataset and thus has none of the distortion characteristic of EBSD. Thus, we can project our data down to two dimensions in many ways depending on the purpose (stereographic for indexing, or orthographic for intuitive visualization). For small grains we still have sufficient SNR to resolve many bands. In addition, the large field of view enabled by a stage-rocked channeling pattern means that every pattern has enough bands (>3) to enable an accurate indexing.

In this work we have only considered grouping of pixels with common orientations into unique grains, but in principle arbitrary subsets of pixels down to the single pixel level can be also be analyzed depending on the length scale of a feature of interest (dislocations, strain, etc.). In addition, the same beam divergence correction we demonstrated for a single crystal sample could be applied here to further improve angular resolution. However, such a correction would be less effective for smaller grains at higher magnifciation and less uniform following spatial drift and distortion correction. In Fig. 4 we present the segmented grain structure maps and several integrated ECPs for each grain, where grain structures from the OMEC ECPs are identical to the segmentation performed using commercial software for EBSD maps. The spatial resolution in our system is limited by the stage accuracy and the ability to maintain a consistent FOV at high magnification. However, given a more precise stage and sufficient contrast (or fiducials) for image alignment, the OMEC ECP resolution should approach that of the BSE image.

As previously mentioned, stage-rocked electron channeling in OMEC allows for a higher angular field of view when compared to beam rocked channeling patterns (70 degrees in OMEC vs 5–10 degrees in beam-rocked ECPs). In fact, the technique should achieve a much higher angular range than demonstrated here, likely greater than 140 degrees given sufficient BSE signal. The large angular field of view allows OMEC ECPs to be directly indexed by traditional Hough-space band detection and triplet voting methods, since we generally have 4+ bands present in each pattern. This avoids the need for specialized indexing methods based on dynamical image simulations and dictionary pattern matching needed to accommodate the limited field of view in beam-rocked ECPs. Also, no specialized distortion correction to account for the tilted gnomonic geometry of a standard EBSD pattern is necessary to correctly index the orientation. Since OMEC produces true three-dimensional orientation maps, the band angles can be compared directly in either stereographic projection, orthographic projection, or in three-dimensional space, potentially leading to more accurate indexing.

One limitation of OMEC is the low-throughput nature of imaging, since each mapping involves the recording of thousands of individual ECCI BSE images at different stage orientations. Although a higher sensitivity BSE detector could substantially reduce the total collection time, the sheer number of orientations that must be probed is a major complication. Certainly, data collected in this *brute force* manner is oversampled, as not every stage tilt/rotation contributes useful of orientation information. For example, space between the primary bands is effectively blank and is wasted sampling time. Ideally, we would want to infer the tilts/rotations which contain information about major bands and their angles with respect to one another, since this is the primary information we use for indexing a crystal’s orientation. Fortunately, substantial progress has been reported on computationally efficient dynamic (adaptive) sampling methods^34,35^. After each measurement, a crude estimation of the object’s channeling pattern can be constructed, and areas of potentially useful content predicted from features of the crude pattern. Since bands have well defined patterns of contrast due to diffraction, it is not difficult to construct a library of typical features to train a supervised algorithm to make a prediction about the next best measurement. Once trained, the algorithm can then construct crude estimates and make new predictions iteratively as it incorporates information obtained from the last measurement.

**Figure 5:**
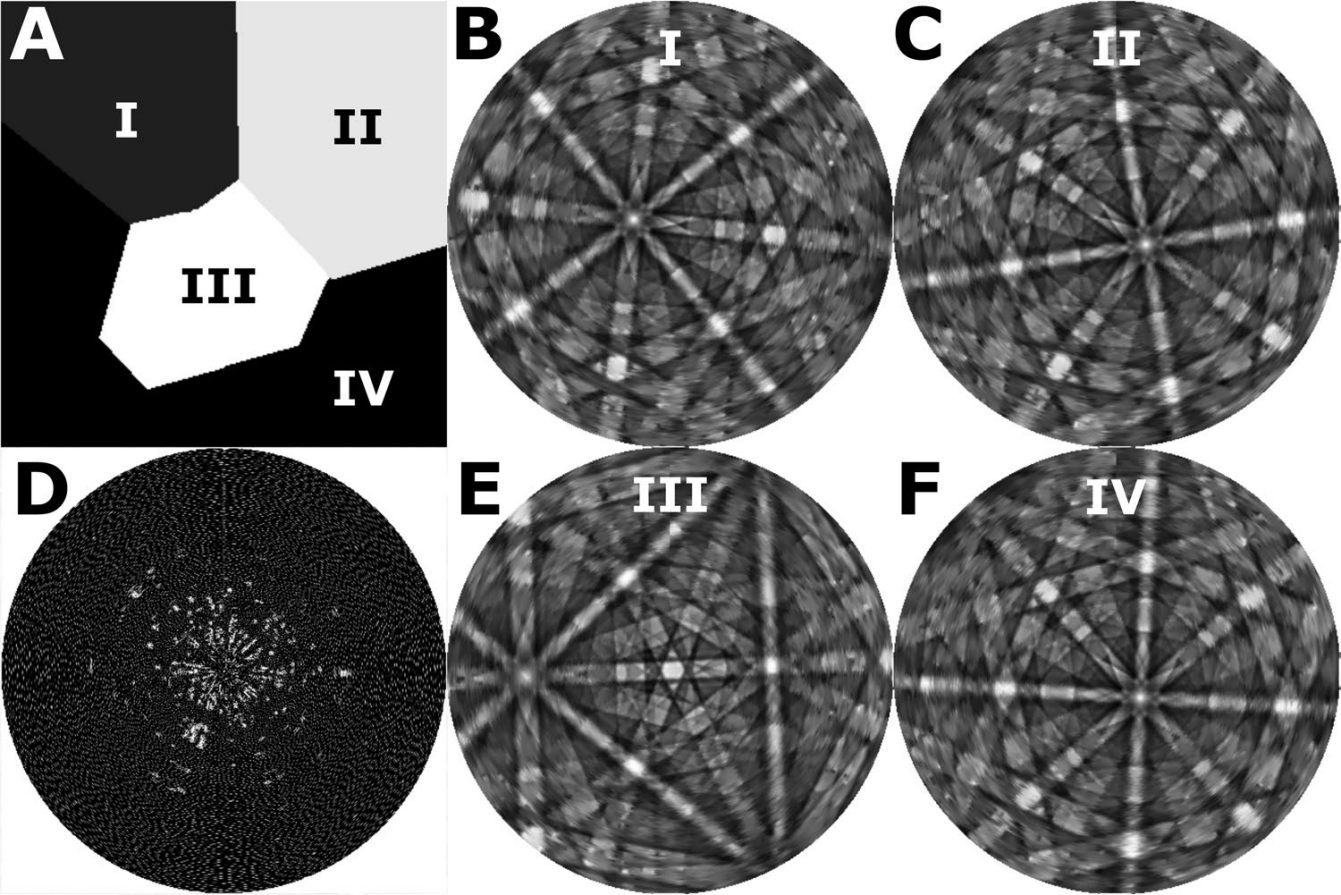
Simulated OMEC acquisition from calculated channeling patterns of Austenite. (**A**) Simulated BSE image at 0 degree tilt and 0 degree rotation for the four reconstructed orientations in (**B**,**C**,**E**,**F**). When choosing which tilts/rotations to sample, the dynamic sampling approach chooses the point with the most benefit for all four grains simultaneously (multi-objective). **(B**,**C**,**E**,**F)** Reconstructed channeling patterns for four differently oriented Austenitic grains in a polycrystalline microstructure after sampling 10% of the possible tilts/rotations using dynamic sampling. (**D**) Mask of selected tilts/rotations, white representing a sample position where a simulated BSE image was recorded, and black representing skipped.

Figure 5 shows a simulated example of how OMEC might benefit from using dynamic sampling. Using multi-beam electron simulations^33^ we simulate a small four grain polycrystalline microstructure with several disparately oriented channeling patterns, parameterized by by tilt (φ) and rotation (θ). After observing a measurement, the algorithm updates the estimation of the true pattern and proposes the next measurement which will have the largest positive effect on the pattern quality. At a sampling rate of only 10% for the simulated OMEC acquisition, the patterns are easily distinguishable, with many indexable bands well resolved. Particularly, prioritizing measurements on the edges of bands allows indexing to be performed very early on during the acquisition process, and balancing the acquisition of points between grains with differing orientation contrast is manageable A ~90% time savings for OMEC orientation mapping potentially brings the time to complete a map into the typical time range of an EBSD map.

Given the volume of data generated by a single OMEC experiment (thousands of spatially correlated ECCI BSE images), significant potential for data mining/engineering exists. For complicated specimens with multiple defects existing simultaneously, OMEC may facilitate disentanglement of multiple microstructural elements, since we have effectively collected an ECCI at every possible orientation. Multiple two-beam-like conditions can be compared offline without the need to identify an orientation on-the-fly using a correlative technique, as some defects may only be visible at specific orientations that would otherwise be missed. OMEC also certainly benefits from the potential of dynamic-sampling by prioritizing measurements at the intersection or edges of bands, thus oversampling the desirable information for ECCI analysis while ‘synthesizing’ the missing ECCIs based on redundant information (potentially taking advantage of both spatial and orientation space redundancies).

### Summary and Conclusions

We have developed a new crystal orientation mapping method, OMEC, combining advantages of ECCI and SACP imaging, which enables orientation mapping over a large FOV with high angular range and distortion-free projection. We have demonstrated the method with up to seventy-degree angular imaging with micron-scale resolution, but much larger angular FOV and spatial resolution should be accessible with a more sensitive BSE detector and higher precision stage. In addition, this technique can be directly implemented on a commercial microscope (possessing adequate stage control) without any additional hardware. We expect that difficulties due to the low-throughput nature of imaging may effectively be ameliorated by dynamic or “adaptive” stage control, where the collection of contrast-critical ECCIs is prioritized over orientations with relatively low contrast contribution to the channeling pattern. In addition, the large-data format and multiple interlinked contrast mechanisms characteristic of ECCIs may allow for the imaging of multiple complicated defects simultaneously, opening the quantification of difficult to analyze specimens.

## Materials and Methods

### Sample preparation and data collection

To sample enough orientations to generate a reasonably high resolution ECP, automated imaging was employed. To accomplish this, a script was developed to capture images at a range of rotation and tilt steps on a commercial FIB/SEM (FEI Helios Nanolab) using the manufacturer’s scripting interface. The standard instrument stage has eucentric tilting and rotation capabilities, which were accurate enough for this application. A typical experiment included 0.5 degree tilt steps from 0 to 34.5 degrees (69 degrees angular range) and 2 degrees rotation steps from 0 to 358 degrees, resulting in a 12,600 image data set (512 × 442 px) over approximately 10 hours. Imaging was accomplished with a 2–5 kV accelerating voltage, high beam current (1.4–22 nA) and the TLD in BSE mode. Silicon samples were imaged without any surface preparation, but the mechanically polished PbSe/GeSe sample was cleaned *in situ* with a plasma cleaner to remove organics (iBss GV10x) followed by a 5 kV Ga ion beam to remove surface oxidation.

### Single crystal silicon data processing

ECP’s are generated from the SEM images by plotting the average contrast of the entire image or a subset of the image (grain, etc.) as a spherical projection according to the beam-to-sample orientation. These data are displayed as an orthographic or stereographic projection but are manipulated in spherical coordinates. There is systematic contrast variation in these images according to the sample tilt condition which is corrected by a background subtraction at each tilt step based on the average contrast for all rotation steps. As the beam is deflected during raster scanning, the beam diverges from the nominal beam-to-sample orientation. This results in a different orientation condition for every pixel in the SEM image, which depends on the working distance, pixel position and sample tilt. For the silicon samples, the background-corrected 512 × 442 pixel images were cropped and binned to generate 16 × 16 pixel subsets and a divergence correction was applied to plot the average contrast value of each subset. This was accomplished by assigning a vector to each nominal orientation position and performing a Rodrigues rotation about that vector for the divergence associated with each subset. As the divergence angles during raster scanning are proprietary and not known to the authors, we estimated these angles empirically to improve ECP quality. The resulting data set was adjusted for contrast variation due to beam contamination by subtracting the intensity of a 3^rd^ degree polynomial fitted to the data in order of collection. During the final display operation, overlapping data points at low tilts were handled by grid-cell averaging and gaps between data points at high tilts were interpolated.

### Polycrystalline sample data processing

For polycrystalline samples, an image sequence of tilted/rotated BSE images was collected and then transferred offline to a processing computer for image alignment/correction. Briefly, the image stack is composed of multiple tilted view of an approximately flat plane from a point illumination source. Our goal will be to bring all different viewpoints on to a common coordinate frame to account for any errors in stage movement and to correct the projected distortion from acquiring a three-dimensional motion onto a two-dimensional grid. Misalignments in the three-dimensional motion of the stage can cause unwanted rotations and translations if they occur in the two-dimensional plane perpendicular to the electron optics, or scaling/magnifications if they occur parallel to electron optics. In addition, since the plane over which the beam scans remains fixed relative to the stage, three-dimensional rotation of the sample around its eucentric point will cause distortion. To reconstruct a pixel-by-pixel or grain-by-grain orientation map, an alignment method must sufficiently correct for the artifacts while being contrast independent (since grain contrast inherently changes between angular frames).

Given a few accurate points of correspondence between two images of planar objects captured at different perspectives, we can define a homography matrix *H* to warp the remaining pixels onto a common coordinate frame. Algorithms to detect keypoint features of correspondence between images at different perspectives and illuminations, such as the Scale Invariant Feature Transform (SIFT)^36^, can be used to define this set of corresponding points. For image sequences collected of polycrystalline specimens in OMEC, SIFT keypoint detection followed by a homography based image warp is sufficient to bring all orientations onto a common spatially correspondent coordinate frame. Once a common set of coordinates is established, an orientation map for each pixel can be produced exactly as described above in the case of the single crystal silicon specimen for arbitrary or defined subsets of pixels with shared orientations.

After image alignment, differences in illumination due to geometric variation in detection efficiency are subtracted using a rolling ball filter with a large radius or local contrast equalization. Finally, the entire dataset can be segmented into grains by either directly clustering the orientation space data at each pixel or by summing the contrast over the entire aligned dataset and applying an edge detector. Each set of pixels corresponding to a common orientation is then summed to produce a high signal-to-noise ratio (SNR) pattern. In microscopes with dedicated back-scatter detector with high detector efficiency, it’s likely that this summation step will be unnecessary as each pattern will have sufficient SNR for interpretation.

EBSD analysis for reference was carried out on an FEI Quanta 650 FEG with an Oxford/HKL EBSD system. Maps were generated at 20 kV and EBSP collection was carried out at 5 kV to correspond more closely to the electron channeling data.

### Dynamic Sampling

For each training experiment the algorithm under-samples the image, estimates the true image, and extracts features and utility estimates for each un-sampled pixel. A vector of coefficients then maps these features to a utility estimate, which is trained using many under-sampling conditions offline. These trained coefficients are used to iteratively predict the best measurement locations for the stage tilt/rotation commands for the crystal. To perform our simulation, each orientation map is divided into several sampling trajectories parameterized by tilt (φ) and rotation (θ). The algorithm makes a prediction and observes one pixel from the true image, from which it infers a future predicted “best” measurement. This proceeds until 10% of the total pixels have been measured. The dynamic sampling algorithm was implemented as described by Godaliyadda, *et al*.^34^. Extending the single-objective dynamic sampling algorithm to multiple different mapping objectives (polycrystals) is trivial. Each grain is considered separately, and the total reduction in distortion for each point is taken as the mean reduction in distortion across all grains. For training data, a single ECP of Silicon (100) was used.

The datasets generated during and/or analyzed during the current study are available from the corresponding author on reasonable request.
